# Only Acyl Carrier Protein 1 (AcpP1) Functions in *Pseudomonas aeruginosa* Fatty Acid Synthesis

**DOI:** 10.3389/fmicb.2017.02186

**Published:** 2017-11-10

**Authors:** Jin-Cheng Ma, Yun-Qi Wu, Dan Cao, Wen-Bin Zhang, Hai-Hong Wang

**Affiliations:** ^1^Guangdong Provincial Key Laboratory of Protein Function and Regulation in Agricultural Organisms, College of Life Sciences, South China Agricultural University, Guangzhou, China; ^2^Department of Chemistry and Biomolecular Sciences, Macquarie University, North Ryde, NSW, Australia; ^3^Forensic Science Center of Qingyuan, Qingyuan Public Security Department, Qingyuan, China

**Keywords:** *Pseudomonas aeruginosa*, fatty acid biosynthesis, acyl carrier protein

## Abstract

The genome of *Pseudomonas aeruginosa* contains three open reading frames, PA2966, PA1869, and PA3334, which encode putative acyl carrier proteins, AcpP1, AcpP2, and AcpP3, respectively. In this study, we found that, although these apo-ACPs were successfully phosphopantetheinylated by *P. aeruginosa* phosphopantetheinyl transferase (PcpS) and all holo-forms of these proteins could be acylated by *Vibrio harveyi* acyl-ACP synthetase (AasS), only AcpP1 could be used as a substrate for the synthesis of fatty acids, catalyzed by *P. aeruginosa* cell free extracts *in vitro*, and only *acpP1* gene could restore growth in the *Escherichia coli* *acpP* mutant strain CY1877. And *P. aeruginosa* *acpP1* could not be deleted, while disruption of *acpP2* or *acpP3* in the *P. aeruginosa* genome allowed mutant strains to grow as well as the wild type strain. These findings confirmed that only *P. aeruginosa* AcpP1 functions in fatty acid biosynthesis, and that *acpP2* and *acpP3* do not play roles in the fatty acid synthetic pathway. Moreover, disruption of *acpP2* and *acpP3* did not affect the ability of *P. aeruginosa* to produce *N*-acylhomoserine lactones (AHL), but replacement of *P. aeruginosa* *acpP1* with *E. coli* *acpP* caused *P. aeruginosa* to reduce the production of AHL molecules, which indicated that neither *P. aeruginosa* AcpP2 nor AcpP3 can act as a substrate for synthesis of AHL molecules *in vivo*. Furthermore, replacement of *acpP1* with *E. coli* *acpP* reduced the ability of *P. aeruginosa* to produce some exo-products and abolished swarming motility in *P. aeruginosa*.

## Introduction

Members of the superfamily of 4′-phosphopantetheine (Ppant)-dependent carrier proteins play central roles in fatty acid synthesis, polyketide synthesis and non-ribosomal peptide synthesis (Lai et al., 2006; Smith, 2006; Byers and Gong, 2007; Chan et al., 2009). This family includes the acyl carrier proteins (ACP), aryl carrier proteins and peptidyl carrier proteins, which can exist as a domain within large multifunctional fatty acid synthase (FAS) or polyketide synthase (PKS) proteins (type I), or as a small monomeric protein in the system for fatty acid biosynthesis in bacteria and plastids (type II FAS) and in the dissociated PKSs (type II PKS) (White et al., 2005; Lai et al., 2006; Smith, 2006; Byers and Gong, 2007; Chan et al., 2009). In the bacterial fatty acid biosynthetic pathway, ACP is a small (molecular mass < 10 kDa), acidic (isoelectric point < 4) protein, which acts as a carrier of acyl intermediates (White et al., 2005). ACP is synthesized in the inactive apo form, which must be converted to the active holo form by a holo-ACP synthase (AcpS) that transfers the 4′-phosphopantetheine from coenzyme CoA (CoA) to a conserved serine residue of apo-ACP (Lambalot et al., 1996; Flugel et al., 2000). The fatty acyl group is linked to ACP by a thioester bond, in which the thiol of the 4′-phosphopantetheine prosthetic group in ACP is condensed with the carboxyl group of the fatty acid (White et al., 2005).

In *Escherichia coli*, there is a single and absolutely essential ACP, which is involved not only in the synthesis of fatty acyl chains but also in their transfer during phospholipid, lipid A or haemolysin synthesis (Byers and Gong, 2007). However, bacteria with complex metabolism often have additional ACPs or ACP homologs that are involved the synthesis of specialized fatty acids, polyketides or non-ribosomal peptides (Lai et al., 2006; Byers and Gong, 2007; Chan et al., 2009). *Sinorhizobium meliloti* has at least six ACPs, and four of them have been well characterized (Geiger and Lopez-Lara, 2002): AcpP for general fatty acid synthesis (López-Lara and Geiger, 2000), NodF for the synthesis of polyunsaturated fatty acids in nodulation factors (Demont et al., 1993; Demont-Caulet et al., 1999), AcpXL for the synthesis and transfer of 27-hydroxyoctacosanoic acid during rhizobial lipid A synthesis (Brozek et al., 1996; Haag et al., 2011), and RkpF, which participates in the synthesis of rhizobial capsular polysaccharides (Epple et al., 1998). The remaining two ACP homologs, SMc01553 and SMb20651, also have been identified as ACP-like proteins, but their functions need further investigation (Ramos-Vega et al., 2009; Davila-Martinez et al., 2010).

*Pseudomonas aeruginosa* is an opportunistic pathogen in humans, and is associated with infections in burn patients and in the respiratory tracts of patients with cystic fibrosis (Driscoll et al., 2007; Moore and Flaws, 2011). In *P. aeruginosa*, fatty acids play a multifaceted role in both maintaining bacterial viability and virulence (Hoang et al., 2002; Zhu and Rock, 2008; Yuan et al., 2012). Aside from being compulsory components of membrane phospholipids, fatty acids are utilized by multiple primary and secondary metabolic pathways in *P. aeruginosa*, including the formation of lipopolysaccharides (LPS) (Raetz and Whitfield, 2002), the synthesis of two important metabolic enzyme cofactors, lipoate and biotin (Spalding and Prigge, 2010), the production of three acylated quorum-sensing (QS) signal molecules—pseudomonas quinolone signal (PQS), N-(3-oxodo-decanoyl)-L-homoserine lactone (3-oxo-C_12_-HSL), and *N*-butanoyl-L-homoserine lactone (C_4_-HSL) (Jimenez et al., 2012)—and the assembly of two virulence factors, siderophore pyoverdine (Hannauer et al., 2012) and rhamnolipids (Zhu and Rock, 2008). Therefore, the fatty acid biosynthetic pathway in *P. aeruginosa* is believed to be an attractive target for the development of new antimicrobial agents.

The *P. aeruginosa* genome contains three open reading frames annotated as encoding ACP (Murugan et al., 2010). PA2966 (now named *acpP1*) encodes AcpP1, which is found in the fatty acid biosynthetic gene cluster (Kutchma et al., 1999). PA1869 (now named *acpP2*) is the second ACP-encoding gene, and PA3334 (now named *acpP3*), which encodes the third ACP, is located in a large gene cluster, and is considered to be involved in the synthesis of an unknown secondary metabolite (Quadri et al., 1999). Although, *in vitro*, these three apo-ACPs have been successfully phosphopantetheinylated by *E. coli* holo-ACP synthase and all holo-forms of these three proteins can be acylated using chemical methods (Raychaudhuri et al., 2005), there is little known about their functions *in* *vivo*. Previous studies showed that *P. aeruginosa* LasI used AcpP1 as a substrate to produce 3-oxo-C_12_-HSL (Hoang et al., 2002) and that AcpP2 was a good substrate for RhlI in the synthesis of C_4_-HSL (Raychaudhuri et al., 2005), which suggests that these three ACPs may have different functions in *P. aeruginosa*. However, there is a lack of *in* *vivo* evidence to support the above hypothesis.

In this study, we constructed *P. aeruginosa acpP2* and *acpP3* mutant strains by insertion of a gentamicin resistance cassette into their ORFs, respectively, and an *acpP1* mutant strain by replacement of the *acpP1* gene with *E. coli* *acpP*. We analyzed various exo-products (including rhamnolipids, siderophore, and three quorum-sensing signal molecules) and tested a selection of virulence factors in the above mutant strains. The results confirmed that only AcpP1 plays roles in the production of these exo-products and virulence factors.

## Materials and Methods

The supply sources were as follows: malonyl-CoA, acetyl-CoA, fatty acids, cerulenin, NADH, NADPH, and antibiotics were obtained from Sigma (St. Louis, MO, United States). Takara Biotechnology (Dalian, China) provided molecular biology reagents. Novagen (Madison, WI, United States) provided pET vectors. Invitrogen (Carlsbad, CA, United States) provided the Ni^2+^-agarose column. Bio-Rad (Hercules, CA, United States) provided the Quick Start Bradford dye reagent. All other reagents were of the highest available quality.

### Bacterial Strains, Plasmids, and Growth Media

The *E. coli* K-12 strains, *P. aeruginosa* strains, and plasmids used in this study are listed in Supplementary Table S1. Luria–Bertani (LB) medium was used as the enrichment medium for growth of *E. coli* and *P. aeruginosa*. *Chromobacterium violaceum* CV026 and *Agrobacterium tumefaciens* NTL4 (pZLR4) were grown on LB medium at 28°C. Antibiotics were used at the following concentrations (in micrograms per milliliter): sodium ampicillin, 100; kanamycin sulfate, 30; chloramphenicol, 30; and gentamicin, 10 (for *E. coli*) or 100 (for *P. aeruginosa*). L-Arabinose was used at a final concentration of 0.01%. Isopropyl-β-D-thiogalactoside (IPTG) was used at a final concentration of 1 mM, and 5-bromo-4-chloro-3-indolyl-β-D-galactoside (X-Gal) was used at a concentration of 20 μg/mL.

### Recombinant DNA Techniques and Construction of Plasmids

To clone the *P. aeruginosa acpP1*, *acpP2,* and *acpP3* genes, genomic DNA was extracted from *P. aeruginosa* strain PAO1 using the Takara DNA extraction kit. The PCR products, amplified from strain PAO1 genomic DNA using *Pfu* DNA polymerase and the primers listed in Supplementary Table S2, were digested by NdeI and HindIII and inserted into pBAD24m (Zhu et al., 2010) between the same sites to produce plasmids: pBAD24m-*acpP1*, pBAD24m-*acpP2,* and pBAD24m-*acpP3*. The *acpPs* genes were confirmed by sequencing by Shanghai Sangon, Inc. (Shanghai, China).

To produce plasmids pCD4 or pCD5, the PCR fragments that were amplified from plasmids pBAD24m-*acpP1* or pBAD24m-*acpP2*, using the primers listed in Supplementary Table S2, were digested with XbaI and HindIII and cloned into pET-28 (b) at the same sites. The *acpP3* fragment of pBAD24m-*acpP3* digested by NdeI and HindIII was cloned between the NdeI and HindIII sites of pET-30 (a) to yield plasmid pCD6. To construct plasmids pCD8, pCD9, and pCD10, the plasmids pCD4, pCD5, and pCD6 were digested with XbaI and HindIII, respectively, and the DNA fragments were purified and inserted into the same sites of pBluescript SK(+), respectively. To yield plasmids pCD1, pCD2, and pCD3, the PCR fragments were amplified from plasmids pCD8, pCD9, or pCD10 using the primers listed in Supplementary Table S2, respectively, and then digested with double restriction enzymes, for which the sites had been designed into the primer sequences. After purification, the fragments were ligated with the vector pTac85 (Marsh, 1986), and they were digested with the same restriction enzymes, respectively.

### Expression and Purification of Plasmid-Encoded Proteins

The pET-28b-derived plasmids pCD4, pCD5, and pCD6 were introduced into *E. coli* strain BL21 (DE3), and the respective proteins, PaAcpP1, PaAcpP2, and PaAcpP3, were expressed at high levels and purified as described previously (Thomas and Cronan, 2005). The plasmids pYFJ84 and pCD15 were introduced into *E. coli* strain BL21 (DE3) for expression of *Vibrio harveyi* AasS and *P. aeruginosa* PcpS proteins. The AasS and PcpS proteins were also purified as described previously (Barekzi et al., 2004; Jiang et al., 2006).

### Phosphopantetheinylatation and Acylation of *P.* *aeruginosa* ACPs

The form of the ACPs was detected by measuring their migration pattern using conformationally sensitive gel electrophoresis. Briefly, the standard assay mixtures contained, in a volume of 40 μL, 0.1 M Tris-HCl (pH 8.8); 1 mM CoA, 25 mM MgCl_2_, 2 mM DTT, 100 μM *P. aeruginosa* ACPs, and 2 μg *P.* *aeruginosa* PcpS. The mixture was pre-incubated at 37°C for 1 h. The reaction products were resolved by conformationally sensitive gel electrophoresis on 17.5% polyacrylamide gels containing 0.5 M urea optimized for separation. For purification of *P.* *aeruginosa* holo-ACPs, 2 mL of the above reaction mixture was used and incubated at 37°C for 4 h. The purification of holo-ACPs was carried out according to the methods described previously (Zhao et al., 2005). Briefly, two volumes of acetone were added to the reaction mixture, and the mixture was allowed to precipitate overnight at -20°C. The precipitates were pelleted at 20,000 × *g* for 20 min at 4°C and then washed twice with three volumes of acetone. The pellet was air-dried and re-suspended in 200 μL Tris-HCl (20 mM, pH 7.4), and the suspension was centrifuged at 20,000 × *g* for 30 min at 4°C. The supernatants were collected and stored at -80°C. Their molecular masses were determined by matrix-assisted laser desorption/ionization (MALDI)-time-of-flight (TOF)-mass spectrometry (MS) (Bruker Autoflex III; Freemont, CA, United States) according to the methods described previously (Mao et al., 2015).

For acylation of *P.* *aeruginosa* holo-ACPs, we used the protocols described by Jiang et al. (2006). The reaction mixture contained, in a volume of 40 μL, 10 mM ATP, 20 mM MgSO_4_, 0.1 M Tris-HCl (pH 7.8), 1 mM dithiothreitol, 0.1 mg *P.* *aeruginosa* holo-ACPs, 0.5 mM octanoic acid and 4 μg *V. harveyi* AasS. The reaction mixtures were incubated at 37°C for 2 h and resolved by conformationally sensitive gel electrophoresis on 17.5% polyacrylamide gels containing 2.5 M urea optimized for the separation.

### Reconstruction of *P. aeruginosa* Fatty Acid Synthesis *in Vitro*

Cell-free extracts of *P. aeruginosa* were prepared according the processes described previously (Hoang and Schweizer, 1999). The exponentially growing cells (OD_600_ of 0.8 to 1.0) in 100 mL LB medium were harvested by centrifugation and then suspended and washed in 2 mL 0.1 M sodium phosphate buffer (pH 6.5). Cell lysates were prepared by passing the cell suspensions three times through a French pressure cell (19,000 lb/in^2^). Cell debris was removed by ultracentrifugation for 1 h at 200,000 × *g*, and the supernatants were saved as cell extracts. The protein concentration of the cell extract was determined using the Quick Start Bradford dye reagent. The fatty acid synthetic reaction mixtures included, in a volume of 100 μL, 0.1 M Tris-HCl (pH 7.8), 1 mM dithiothreitol, 100 μM *P. aeruginosa* holo-ACPs (AcpP1, AcpP2, or AcpP3), 100 μM NADH, 100 μM NADPH, 100 μM malonyl-CoA, 100 μM acetyl-CoA and 5 mg *P. aeruginosa* cell-free extract. Reactions were incubated at 37°C for 2 h. Analysis of the ACP thioesters was performed by conformationally sensitive gel electrophoresis on 17.5% polyacrylamide gels containing 2.5 M urea optimized for the separation. Cerulenin was added to the reaction mixtures at a final concentration of 100 μM when needed.

### Mutation of *P. aeruginosa* *acpPs* Genes

To disrupt the *P. aeruginosa acpPs* genes, suicide plasmids were constructed as follows. The 1.3-kb DNA fragment containing *acpP1* amplified from *P. aeruginosa* genomic DNA using the primers listed in Supplementary Table S2 was digested with BamHI and HindIII and cloned between the same sites of pBluescript SK (+) to produce pCD16. A gentamicin resistance cassette amplified from p34s-Gm (Dennis and Zylstra, 1998) using the primers listed in Supplementary Table S2 was digested by EcoRI and inserted into the same site of pCD16 to produce pCD17. Subsequently, the BamHI–HindIII fragment from pCD17 was inserted between the same sites of pK18mobsacB (Schafer et al., 1994) to yield the suicide plasmid pCD18. A similar method was used to construct the suicide plasmids pCD21 and pCD25. All these plasmids were introduced into *P. aeruginosa* PAO1 via conjugal transfer from *E. coli* S17-1 (Supplementary Figure S1A). Single-crossover integrants into the strain PAO1 chromosome were selected by chloramphenicol and gentamicin resistance. Cultures grown from the integrant colonies were plated onto LB medium containing 10% sucrose and gentamicin in order to select for the loss of the vector sequences from the PAO1 chromosome. The successful construction of the designed mutations was evaluated by PCR analysis using the primers listed in Supplementary Table S2. Two single mutant strains were obtained: PA-A2 (*acpP2*::Gm^r^) and PA-A3 (*acpP3*::Gm^r^) (Supplementary Figures S1B,C), but no *acpP1* insertion mutant was obtained.

To replace *P. aeruginosa* *acpP1* with *E. coli acpP*, the DNA fragments acpP1up, EcacpP and acpP1down were amplified using the primers listed in Supplementary Table S2. The above three DNA fragments were ligated by overlap PCR and cloned into the T-vector, pMD19, to produce pCD29. The BamHI–HindIII fragment from pCD29 was cloned between the same sites of pK18mobsacB to yield plasmid pCD30. The gentamicin resistance cassette of p34s-Gm, digested by BamHI, was inserted into the same site of pCD30 to produce plasmid pCD31. Using similar procedures, the replacement strain PA-A1 (*a*c*pP1*::Ec*acpP*) was obtained (Supplementary Figure S3).

To obtain double or triple *P. aeruginosa acpPs* mutant strains, we also constructed plasmid pCD22, in which the gentamicin resistance cassette in plasmid pCD 21 was replaced with a tetracycline resistance cassette (Dennis and Zylstra, 1998). Using similar procedures to those described above, on a background of PA-A1, we obtained the double mutant strains PA-A12 (*a*c*pP1*::Ec*acpP acpP2*::Gm^r^) (Supplementary Figure S4A) and PA-A13 (*a*c*pP1*::Ec*acpP acpP3*::Gm^r^) (Supplementary Figure S4B). On a background of PA-A3, we obtained the double mutant strain PA-A23 (*a*c*pP2*::Tc^r^ *acpP3*::Gm^r^) (Supplementary Figure S4C), and on a background of PA-A23, we produced a triple mutant strain, PA-A123 (*a*c*pP1*::Ec*acpP a*c*pP2*::Tc^r^ *acpP3*::Gm^r^) (Supplementary Figure S4D).

To confirm the mutant strains, all *acpP* allelic genes were sequenced by Shanghai Sangon, Inc. (Shanghai, China).

### Analysis of Phospholipid Composition

The cultures were grown aerobically at 37°C in LB medium overnight. Cells were harvested and washed three times with sterile water. Fatty acid methyl esters were synthesized and extracted as described previously (Stead, 1989). Briefly, cellular lipids were saponified by the addition of 2 mL of sodium hydroxide/methanol solution at 100°C for 40 min with shaking (800 rpm). The fatty acids were then methylated by the addition of 4 mL of hydrochloric acid/methanol solution at 80°C for 30 min, and immediately cooled to below 20°C. Fatty acid methyl esters were obtained by three extractions, each with 1 mL of petroleum ether. The solvent was removed under a stream of nitrogen, and the residue was dissolved in 100 μL of hexane. The crude extract was passed through a 0.22 μm Mini-star filter unit, and 2 μL of the extract was analyzed by gas chromatography–mass spectrometry (GC-MS).

### Motility Assay

The motility assay was carried out as described previously (Huang et al., 2016). The swarming motility of *P. aeruginosa* was investigated using the following media: 0.45% tryptone, 0.13% yeast extract, 0.5% agarose, 0.22% NaCl, and 0.5% agar. The plates were air-dried for 5–10 min before use. The bacterial cells (OD_600_ to 0.8) were gently inoculated using a toothpick at the center of the media surface, and the plates were incubated at 30°C for 24 h. The twitching motility assay was performed using the following media: 1% tryptone, 0.5% yeast extract, 0.5% NaCl, and 1% agarose. The cells were stabbed into the bottom of a Petri dish containing each of the above media using a toothpick, and incubated at 37°C for 20 h. The movement of the colony on the interface between the medium and the dish was observed.

### Rhamnolipid Identification

Rhamnolipids were extracted as previously described (Huang et al., 2016). Briefly, cells were removed from PPGAS (NH_4_Cl 20 mM, KCl 20 mM, Tris-HCl pH 7.2 120 mM, MgSO_4_ 1.6 mM, glucose 0.5%, and peptone 1.0%) cultures grown at 30°C for 24 h by centrifugation (10 min at 6,000 × *g*), and the supernatant was acidified to pH 2 with concentrated HCl. Equal volumes (1 mL) of acidified supernatant and chloroform:methanol (2:1) were mixed and vortexed for 1 min, and the lower organic phase was collected after centrifugation (10 min at 10,000 × *g*). The extraction was repeated, and the pooled organic phases were evaporated to dryness, re-suspended in 1 mL methanol, filtered through a 0.45-μm membrane, evaporated to dryness, and re-suspended in 20 μL methanol. The samples were analyzed by thin-layer chromatography (TLC) using silica 60 F_254_ plates (Merck, Darmstadt, Germany) with a mobile phase consisting of chloroform:methanol:acetic acid (65:15:2, v/v/v). Orcinol reagent dissolved in 15% H_2_SO_4_ at a final concentration of 2% was used to visualize rhamnolipid spots at 100°C for 2–5 min. The concentration of rhamnolipid was determined by measuring the concentration of rhamnose with the sulfuric acid-anthrone reagent (0.2% anthrone, 85% sulfuric acid) method, using rhamnose sugar as the standard, at 620 nm. To identify the fatty acid component of the rhamnolipid, rhamnolipids were extracted from 5 mL supernatant using the same procedures as described above. Fatty acid methyl esters were synthesized using rhamnolipid and extracted as described previously (Zhu et al., 2010). Briefly, the rhamnolipids were dissolved in 1.2 mL dry methanol, and 0.2 mL of 25% (vol/vol) sodium methoxide was added. After the solution was allowed to stand for 15 min at room temperature, 1.2 mL of 2 M HCl was added, and the fatty acid methyl esters were obtained by three extractions each with 1.2 mL of petroleum ether. The solvent was removed under a stream of nitrogen, and the residue was analyzed by measuring the formation of hydroxylacyl-ACP. The standard mixture contained 0.1 M sodium phosphate (pH 7.0), 10 mM ATP, 20 mM MgSO_4_, 1 mM DTT, 100 μM *E. coli* ACP, 1 μg/μL His-tagged *V. harveyi* AasS purified from *E. coli* and fatty acid methyl esters obtained from rhamnolipid. The mixture was incubated at 37°C for 1 h and resolved by conformationally sensitive gel electrophoresis on 17.5% polyacrylamide gels containing 2.5 M urea optimized for the separation.

### Extraction and Assay of Quorum-Sensing Signal Molecules

Extraction of quorum-sensing signal molecules was performed as described previously (Yuan et al., 2012). *P. aeruginosa* cells were grown in LB to the early stationary phase, and cells were removed from 10 mL growth medium by centrifugation at 12,000 × *g* for 15 min at 4°C. Quorum-sensing signal molecules were twice extracted from 10 mL of supernatant for each sample, using either an equal volume of ethyl acetate for homoserine lactones (HSL) or an equal volume of acidified ethyl acetate for 2-heptyl-3-hydroxy-4(1H)-quinolone (PQS). The organic extracts were concentrated to dryness using a nitrogen bubbler, and the residue was resuspended in 100 μL of ethyl acetate (HSL) or methanol (PQS). For determination of AHL-like molecules with short acyl chains, the biosensor *C. violaceum* CV026 was utilized (McClean et al., 1997). *A. tumefaciens* NTL4 (pZLR4) was utilized to detect AHLs with long acyl chains (Cha et al., 1998). HPLC analysis was carried out with an HPLC series (Agilent Technologies, Palo Alto, CA, United States) equipped with an Elite C18 column (4.6 mm × 250 mm, 5 μm particle size) that was maintained at 45°C, with a UV detector set at 250 nm. The mobile phase comprised 0.1% formic acid in water (A) and 0.1% formic acid in acetonitrile (B) (Ortori et al., 2007). The flow rate was 1 mL/min. The elution conditions were as follows: 0 min 35% B, linear gradient to 60% B for 10 min and then a linear gradient from 60 to 95% B over 5 min, then 5 min 95% B and then ramped back to the starting conditions in 9 min. The column was re-equilibrated for a total of 5 min. A 10-μL volume was injected onto the column. PQS content was assessed by normal-phase thin-layer chromatography (TLC) on activated silica 60 F254 plates (Merck) according the method described previously (Yuan et al., 2012). Authentic acyl- and 3-oxo-acyl-HSL, and PQS (Sigma) were used as standards.

### LasA and LasB Activity Assay

*Pseudomonas aeruginosa* cells were grown in LB at 37°C for 12 h, and cells were removed from 10 mL growth medium by centrifugation at 5,000 × *g* for 10 min at 4°C. LasA protease activity was determined by measuring the ability of *P. aeruginosa* culture supernatants to lyse boiled *Staphylococcus aureus* RN4200 cells, as described previously (Huang et al., 2016). A 30-mL overnight culture of *S. aureus* grown in tryptic soy broth was placed in a boiling water bath for 10 min and then centrifuged for 10 min at 10,000 × *g*. The resulting pellet was re-suspended in 10 mM Na_2_HPO_4_ (pH 7.5) and adjusted to an OD_600_ of 0.9. A 100-μL aliquot of bacterial supernatant was then added to 900 μL of *S. aureus* suspension, and the OD_600_ was determined after 5, 10, 15, 20, 25, 30, 35, 40, 45, 60, 75, 90, and 105 min. LasB protease activity in *P. aeruginosa* culture supernatants was determined using the elastin Congo red assay (Yuan et al., 2012). After a 3-h digestion period at 37°C (100 mM Tris, 1 mM CaCl [pH 7.5] with 20 mg of elastin Congo red substrate [Sigma] plus 100 μL of spent supernatant), suspensions were clarified by centrifugation (16,000 × *g*, 5 min, RT), and the absorbance at 495 nm was measured to determine the amount of liberated dye.

### Pyocyanin Quantitation Assay

The pyocyanin assay is based on the absorbance of pyocyanin at 520 nm in acidic solution (Essar et al., 1990). A 5-mL sample of culture grown in LB was extracted with 3 mL of chloroform and then re-extracted into 1 mL of 0.2 N HCl to give a pink to deep red solution. The absorbance of this solution was measured at 520 nm. Concentrations, expressed as μg of pyocyanin produced per mL of culture supernatant, were determined by multiplying the optical density at 520 nm (OD_520_) by 17.072.

### Siderophore Secretion Assay

The CAS-LB plates were prepared according to a modification of the published protocol (Yuan et al., 2012). A 10× blue dye solution consisting of 1 mM chrome azurol S (CAS) (Acros), 2 mM cetyltrimethylammonium bromide, and 500 μM FeCl_3_ 6H_2_O was sterilized by autoclaving, and then 10 mL was added to 100 mL of molten LB Miller agar (1.5%). Plates were allowed to solidify and dried at room temperature for 1 h before streaking cultures containing each strain to be tested.

### Statistical Analysis

An analysis of variance for the experimental datasets was performed using JMP software version 5.0 (SAS Institute Inc., Cary, NC, United States). Significant effects of treatment were determined by the *F*-value (*P* = 0.05). When a significant *F*-test was obtained, a separation of means was accomplished by Fisher’s protected LSD (least significant difference) at *P* = 0.05.

## Results

### Only *P. aeruginosa* AcpP1 Functions in Fatty Acid Biosynthesis

**FIGURE 1:**
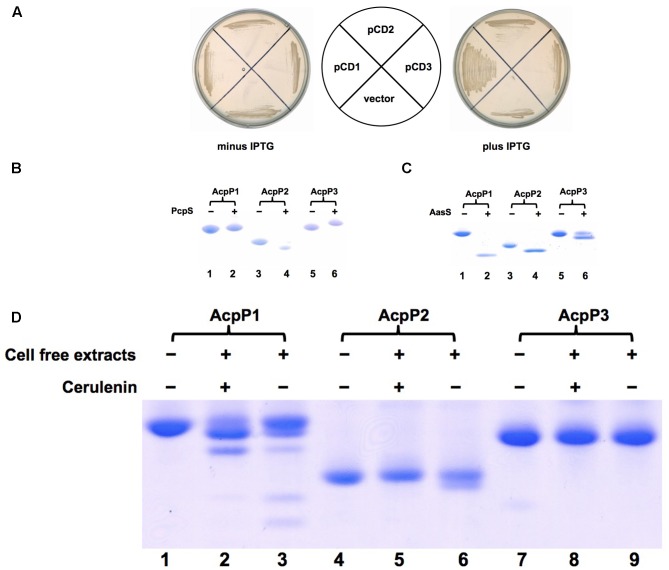
Identification of *Pseudomonas aeruginosa* AcpPs *in vivo* and *in vitro*. **(A)** Complementation of *Escherichia coli acpP* mutant strain CY1877 with *P. aeruginosa* *acpP* genes. *E. coli acpP* mutant strain carried plasmids pCD1, pCD2, pCD3 and empty vector, and was grown on LB plates. pCD1, plasmid pTac 85 carrying *acpP1*; pCD2, plasmid pTac 85 carrying *acpP2*; pCD3, plasmid pTac 85 carrying *acpP3*; vector, pTac 85. **(B)** The *P. aeruginosa* apo-AcpPs were phosphopantetheinylated by *P. aeruginosa* PcpS *in vitro*. *P. aeruginosa* AcpPs were purified from an *E. coli* strain according to the procedures described in section “Materials and Methods.” The reaction mixture containing each kind of AcpP, CoA and *P. aeruginosa* PcpS was incubated for 2 h before being resolved by conformationally sensitive gel electrophoresis as described in section “Materials and Methods.” **(C)** The *P. aeruginosa* holo-AcpPs were acylated by *Vibrio harveyi* AasS *in vitro*. The reaction mixture containing each kind of AcpP, ATP, octanoic acid and *V. harveyi* AasS was incubated for 2 h before being resolved by conformationally sensitive gel electrophoresis as described in section “Materials and Methods”. **(D)** The *P. aeruginosa* AcpPs were used as substrates in fatty acid synthesis catalyzed by *P. aeruginosa* cell-free extracts. The reaction mixture containing each kind of AcpP, acetyl-CoA, malonyl-CoA, NADPH, NADH and cell-free extracts was incubated for 2 h before being resolved by conformationally sensitive gel electrophoresis as described in section “Materials and Methods.”

In order to test whether *P. aeruginosa acpP1*, *acpP2,* and *acpP3* function in fatty acid biosynthesis, these genes were inserted into the IPTG-inducible vector pTac85 (Marsh, 1986) to yield plasmids pCD1 (carrying Pa *acpP1*), pCD2 (carrying Pa *acpP2*), and pCD13 (carrying Pa *acpP3*), respectively. The plasmids were then used to transform *E. coli* strain CY1877, a strain in which the chromosomal *acpP* gene had been deleted and replaced with a chloramphenicol resistance cassette. The deletion replacement event was done in the presence of an ampicillin-resistant pBAD24-derived plasmid expressing the *E. coli* *acpP* gene to allow survival of the *acpP* deletion strain. Growth of strain CY1877 was dependent on the addition of arabinose because transcription of the *acpP* gene was from a vector *araBAD* promoter. Only *E. coli* CY1877 carrying the plasmid encoding *acpP1* (pCD1) grew on LB medium in the presence of IPTG and the absence of arabinose, whereas derivatives of strain CY1877 carrying empty vector, or plasmids encoding *acpP2* or *acpP3* failed to grow under this condition (**Figure 1A**). *P. aeruginosa acpP1* complemented the *E. coli acpP* mutation, showing that *P. aeruginosa* AcpP1 can function as an ACP and replace functional *E. coli* ACP. To examine further whether *P. aeruginosa* AcpP2 and AcpP3 are involved in fatty acid synthesis, these ACPs were overexpressed and purified as described in the section “Materials and Methods.” First, phosphopantetheinylatation of these three ACPs was tested. As expected, after incubation with *P. aeruginosa* phosphopantetheinyl transferase (PcpS) and CoA, the migration of AcpP1 did not change significantly, suggesting that *P. aeruginosa* AcpP1 may be activated from apo-ACP to holo-ACP by *E. coli* holo-ACP synthase (AcpS) (**Figure 1B**), which is consistent with the finding that *acpP1* complemented the *E. coli acpP* mutation. To confirm the above observation, we analyzed AcpP1, which was purified from *E. coli* cells directly or from phosphopantetheinylatation with *P. aeruginosa* PcpS, by MALDI-TOF-MS (Supplementary Figure S5). The data showed that AcpP1 purified from *E. coli* cells includes apo- and holo- forms of ACPs, while after phosphopantetheinylatation with *P. aeruginosa* PcpS, the AcpP1 all became holo-AcpP1 (Supplementary Figures S5A,B). However, both AcpP2 and AcpP3 showed different migration rates after incubation with PcpS and CoA (**Figure 1B**), indicating that the purified AcpP2 and AcpP3 proteins from *E. coli* were apo-ACPs, and that AcpP2 and AcpP3 could not be activated properly in *E. coli* from apo-ACP to holo-ACP by *E. coli* AcpS, which were confirmed by analysis with MALDI-TOF-MS (Supplementary Figures S5C–F). We also tested whether these ACPs can be acylated by *V. harveyi* acyl-ACP synthetase (AasS). After incubation of these holo-ACPs with octanoic acid, ATP and AasS, all reaction mixtures produced new bands on native gels, showing that all these holo-ACPs could be acylated by AasS (**Figure 1C**). Finally, whether these ACPs can be used as substrates during fatty acid synthesis by *P. aeruginosa* *in vitro* was examined. As expected, *P. aeruginosa* AcpP1 was a good substrate for fatty acid synthesis. The reaction mixture containing holo-AcpP1, malonyl-CoA, acetyl-CoA, NADH, NADPH and *P. aeruginosa* cell-free extract produced short-chain acyl-ACPs in the presence of cerulenin, an antibiotic that inhibits the activity of long chain 3-keto-acyl-ACP synthases. In the absence of cerulenin, the reaction mixture produced various long-chain acyl-ACPs (**Figure 1D**). However, *P. aeruginosa* AcpP2 and AcpP3 were poor substrates for fatty acid synthesis *in vitro*: only the reaction mixture containing AcpP2 produced a trace amount of short-chain acyl-ACPs in the absence of cerulenin (**Figure 1D**), but the reaction mixture containing AcpP3 did not produce any acyl-ACPs. All these results imply that *P. aeruginosa* AcpP2 and AcpP3 are not involved in fatty acid synthesis in *P. aeruginosa.*

### *acpP1* Is Essential for *P. aeruginosa* Growth

To examine further the physiological functions of the three *P. aeruginosa* ACP homologs, we attempted to inactivate each of the putative *acpP* genes by allelic replacement. Three pK18mobsacB-borne suicide plasmids used to insert a gentamicin resistance cassette into *acpP1*, *acpP2,* or *acpP3* genes were constructed and yielded pCD18, pCD21, and pCD25, respectively (Supplementary Figure S1). These plasmids were introduced into *P. aeruginosa* PAO1 via conjugal transfer from *E. coli* S17-1 (Supplementary Figure S1). After negative selection, mediated by *Bacillus subtilis* *sacB*, and identification by colony PCR using the primers listed in Supplementary Table S2, two single mutant strains were obtained: PA-A2 (*acpP2*::Gm^r^) and PA-A3 (*acpP3*::Gm^r^) (Supplementary Figures S1A,B), but no *acpP1* insertion mutant was obtained, which indicates that *P. aeruginosa acpP1* seems to be an essential gene.

To confirm this finding, two new suicide plasmids, pCD28 (Supplementary Figure S2) and pCD31 (Supplementary Figure S3A), were constructed. The plasmid pCD28 was used to delete *acpP1*, while pCD31 was used to replace *P. aeruginosa acpP1* with *E. coli* *acpP*. Both plasmids were introduced into *P. aeruginosa* PAO1 by conjugation. Cultures grown from the single-integrant colonies were plated onto LB medium containing sucrose. Colonies that were sensitive to gentamicin were analyzed by PCR using the primers listed in Supplementary Table S2. Unfortunately, no *acpP1* deletion strain was obtained (Supplementary Figure S2). However, we obtained a replacement mutation strain, PA-A1 (*acp1*::*EcacpP*), in which the *P. aeruginosa acpP1* gene was replaced with the *E. coli acpP* gene (Supplementary Figures S3B,C). These results indicated that *acpP1* is essential for the growth of *P. aeruginosa*.

Using similar procedures, we also constructed three double mutants, PA-12 (*acpP1*::*EcacpP*, *acpP2*::Gm^r^) (Supplementary Figure S4A), PA-13 (*acpP1*::*EcacpP*, *acpP3*::Gm^r^) (Supplementary Figure S4B), and PA-23 (*acpP2*::Tc^r^, *acpP3*::Gm^r^) (Supplementary Figure S4C), and one triple mutant, PA-123 (*acpP1::EcacpP*, *acpP2::*Tc^r^, *acpP3*::Gm^r^) (Supplementary Figure S4D). Next, the growth of these mutant strains was tested. All mutant strains were viable and grew as well as wild type strain PAO1 (data not shown).

### Replacement of *acpP1* with *E. coli acpP* Reduced the Ability of *P. aeruginosa* to Produce Quorum-Sensing Signals and Rhamnolipids

To investigate further the functions of the three ACP homologs in fatty acid synthesis in *P. aeruginosa*, the fatty acid composition of the *acpP*s mutant strains grown in LB medium was first determined by gas chromatography–mass spectrometry (GC-MS). The fatty acid profiles of all the mutant strains were almost the same and were not distinct from that of wild type strain PAO1 (**Table 1** and Supplementary Table S3). This indicated that *E. coli* ACP protein may replace functions of *P. aeruginosa* AcpP1, and that AcpP2 and AcpP3 may not play roles in fatty acid synthesis.

**Table 1:** Fatty acid composition of total lipid extracts from *Pseudomonas aeruginosa* strains^a^.

Fatty acid (%)	*P. aeruginosa* strains			
	PAO1	PA-A1	PA-A2	PA-A3
*n*-C_10:0_-3-OH^b^	3.78 ± 0.69	6.01 ± 0.84	4.62 ± 1.99	5.40 ± 0.68
*n*-C_12:0_-3-OH	6.60 ± 0.76	6.59 ± 0.87	6.51 ± 0.23	6.79 ± 1.02
n-C_16:1_	11.26 ± 0.50	10.40 ± 1.51	9.87 ± 1.66	10.83 ± 1.50
n-C_16:0_	34.49 ± 0.36	34.90 ± 0.37	36.10 ± 0.69	36.30 ± 0.83
n-C_18:1_	35.85 ± 1.91	36.12 ± 2.02	36.08 ± 0.68	34.13 ± 1.16
n-C_18:0_	8.01 ± 0.35	5.97 ± 0.72	6.81 ± 0.79	6.55 ± 0.91

^a^*Cells were grown in LB medium for 12 h at 37°C. Total lipids were extracted and transesterified to fatty acid methyl esters, and products identified by GC-MS. Values are percentages of total fatty acids and are means ± standard deviations of three independent experiments. ***^*b*^***n-C_*10:0*_ 3-OH, 3-hydroxyldecanoic acid; n-C_*10:0*_ 3-OH, 3-hydroxyldodecanoic acid; n-C_*16:1*_, *cis*-9-hexadecenoic acid; n-C_*16:0*_, hexadecanoic acid; n-C_*18:1*_, *cis*-11-octadecenoic acid; n-C_*18:0*_, octadecanoic acid.*

In *P. aeruginosa*, fatty acids are not only the compulsory components of membrane phospholipids, but are also utilized to produce exo-products, including three acylated quorum-sensing signal molecules, rhamnolipids and siderophore pyoverdine (Hoang et al., 2002; Zhu and Rock, 2008; Yuan et al., 2012). To confirm the above hypothesis and to determine whether AcpP2 or AcpP3 is specified to synthesize these exo-products, the production of these exo-products in *P. aeruginosa acpP*s mutant strains was examined.

**FIGURE 2:**
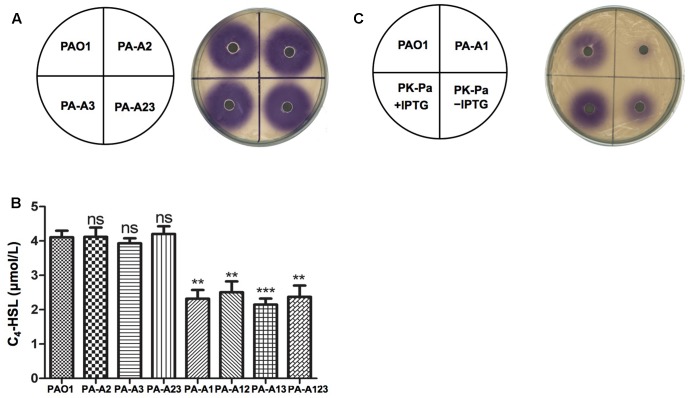
Detection of C_4_-HSL signals produced by *P. aeruginosa* mutant strains. **(A)** Analysis of the C_4_-HSL signals produced by *P. aeruginosa* *acpP2* and *acpP3* mutant strains using *Chromobacterium violaceum* reporter strain CV026. **(B)** Quantitative analysis of the C_4_-HSL signal produced by *P. aeruginosa* *acpPs* mutant strains using high performance liquid chromatography (HPLC). **(C)** Analysis of the C_4_-HSL signals produced by *P. aeruginosa* *acpP1* mutant strains using *C. violaceum* reporter strain CV026. The acylated HSL fraction was extracted from the supernatants of stationary-phase cultures grown in LB broth at 37°C for 12 h. PAO1, *P. aeruginosa* wild type strain; PA-A2, *P. aeruginosa acpP2*::Gm^r^ single mutant strain; PA-A3, *P. aeruginosa* *acpP3*::Gm^r^ single mutant strain; PA-A23, *P. aeruginosa* *acp2*::Tc^r^ *acp3*::Gm^r^ double mutant strain; PA-A1, *P. aeruginosa acp1*::Ec *acpP* single mutant strain; PA-A12, *P. aeruginosa acp1*::Ec *acpP* *acp2*::Gm^r^ double mutant strain; PA-A13, *P. aeruginosa acp1*::Ec *acpP* *acp3*::Gm^r^ double mutant strain; PA-A123, *P. aeruginosa acp1::*Ec *acpP acp2::*Tc^r^ *acp3::*Gm^r^ triple mutant strain; PK-Pa, *P. aeruginosa* *acp1*::Ec *acpP* single mutant strain carrying plasmid, pSRKPa, encoding wild type *P. aeruginosa acpP1* gene. Data are the mean ± standard deviation of triplicate measurements. Pair-wise comparisons were made between wild-type strain PAO1 and each mutant strain by Student’s *t*-test. ^∗∗∗^Highly significant difference, *P* < 0.01. ^∗∗^Significant difference, *P* < 0.05. ns, no significant difference, *P* > 0.1.

First, the *rhl* QS signal molecule, *N*-butanoyl-L-homoserine lactone (C_4_-HSL), produced by *P. aeruginosa acpP*s mutant strains was tested using the *C. violaceum* reporter strain CV026, which produces a purple halo in response to acyl-HSLs. The purple halo around mutant strains PA-A2, PA-A3, and PA-A23 was as large as that around wild type strain PAO1 (**Figure 2A**). The amount of C_4_-HSL produced by PA-A2, PA-A3, or PA-A23 was also determined by high performance liquid chromatography (HPLC), which showed that all these mutant strains produced almost the same amount of C_4_-HSL (about 4.1 μM), which was similar to the amount produced by the PAO1 strain (**Figure 2B**). However, the purple halo around mutant strain PA-A1 was weak and small in comparison with that of wild type strain PAO1 (**Figure 2C**). The HPLC analysis showed that the amount of C_4_-HSL produced by PA-A1 (about 2.4 μM) was significantly lower than that produced by the PAO1 strain (about 4.1 μM) (*P* < 0.01) (**Figure 2C**). In contrast, the double mutant strains, PA-A12 and PA-A13, and the triple mutant, PA-123, produced a similar amount of C_4_-HSL to the PA-A1 strain (**Figure 2B**). However, expression of the wild-type *P. aeruginosa acpP1* gene in mutant strain PA-A1 increased the production of C_4_-HSL, and under IPTG induction the amount of C_4_-HSL was restored to the level of the wild type strain (**Figure 2C**).

**FIGURE 3:**
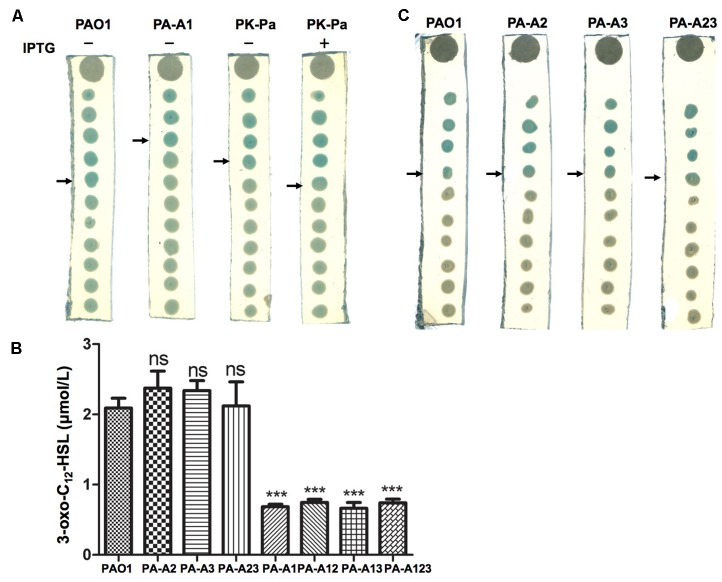
Detection of 3-oxo-C_12_-HSL signals produced by *P. aeruginosa* mutant strains. **(A)** Analysis of the 3-oxo-C_12_-HSL signals produced by *P. aeruginosa* *acpP1* mutant strains using *Agrobacterium tumefaciens* reporter strain NTL4 (pZLR4). **(B)** Quantitative analysis of the 3-oxo-C_12_-HSL signals produced by *P. aeruginosa* *acpPs* mutant strains using high performance liquid chromatography (HPLC). **(C)** Analysis of the 3-oxo-C_12_-HSL signals produced by *P. aeruginosa* *acpP2* and *acpP3* mutant strains using *A. tumefaciens* reporter strain NTL4 (pZLR4). The acylated HSL fraction was extracted from the supernatants of stationary-phase cultures grown in LB broth at 37°C for 12 h. PAO1, *P. aeruginosa* wild type strain; PA-A2, *P. aeruginosa acpP2*::Gm^r^ single mutant strain; PA-A3, *P. aeruginosa* *acpP3*::Gm^r^ single mutant strain; PA-A23, *P. aeruginosa* *acp2*::Tc^r^ *acp3*::Gm^r^ double mutant strain; PA-A1, *P. aeruginosa acp1*::Ec *acpP* single mutant strain; PA-A12, *P. aeruginosa acp1*::Ec *acpP* *acp2*::Gm^r^ double mutant strain; PA-A13, *P. aeruginosa acp1*::Ec *acpP* *acp3*::Gm^r^ double mutant strain; PA-A123, *P. aeruginosa acp1::*Ec *acpP acp2::*Tc^r^ *acp3::*Gm^r^ triple mutant strain; PK-Pa, *P. aeruginosa* *acp1*::Ec *acpP* single mutant strain carrying plasmid, pSRKPa, encoding wild type *P. aeruginosa acpP1* gene. The arrow indicates the diffusion front of 3-oxo-C_12_-HSL. Data are the mean ± standard deviation of triplicate measurements. Pair-wise comparisons were made between wild-type strain PAO1 and each mutant strain by Student’s *t*-test. ^∗∗∗^Highly significant difference, *P* < 0.01. ^∗∗^Significant difference, *P* < 0.05.

The *las* QS signal molecule, *N*-(3-oxododecanoyl)-L-homoserine lactone (3-oxo-C_12_-HSL), produced by these mutant strains was also investigated using the reporter strain *A. tumefaciens* NL4(pZLR4) and HPLC. The data showed that replacement of *acpP1* with *E. coli acpP* reduced the ability of *P. aeruginosa* to produce 3-oxo-C_12_-HSL (**Figures 3A,B**), but mutation of *acpP2* or *acpP3* did not affect the production of the *las* QS signal molecule (**Figures 3B,C**).

We analyzed the levels of PQS in supernatant extracts of the mutant strains by thin-layer chromatography (TLC). The level of PQS was reduced 40% in the supernatant extract of mutant PA-A1 in comparison with that produced by wild type strain PAO1. However, disruption of *acpP2* or *acpP3* did not cause reduction of the amount of PQS produced by *P. aeruginosa* strains (data not shown).

**FIGURE 4:**
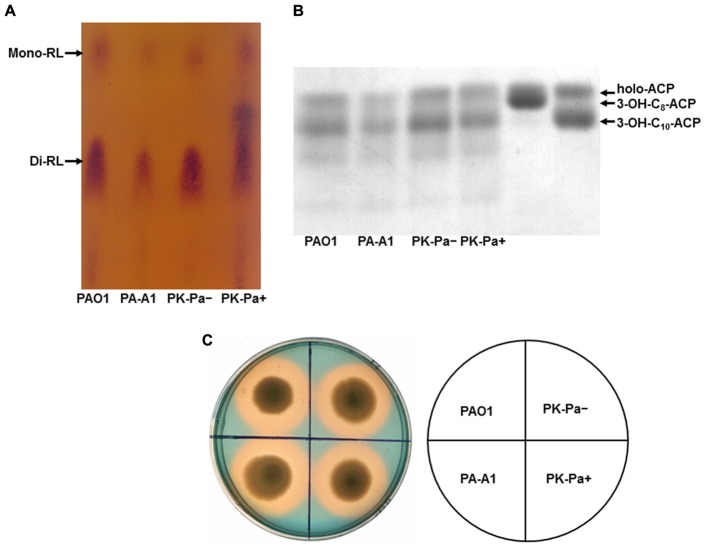
Characterization of rhamnolipid production and siderophore secretion by *P. aeruginosa acpP1* mutant strains. **(A)** Analysis of rhamnolipids produced by *acpP1* mutant strains using thin-layer chromatography (TLC). **(B)** Composition of 3-hydroxyacids in rhamnolipids produced by *acpP1* mutant strains. **(C)** Siderophore secretion in *P. aeruginosa acpP1* mutant strains. PAO1, *P. aeruginosa* wild type strain; PA-A1, *P. aeruginosa* *acp1*::Ec *acpP* single mutant strain; PK-Pa^-^, *P. aeruginosa* *acp1*::Ec *acpP* single mutant strain carrying plasmid, pSRKPa, encoding wild type *P. aeruginosa* *acpP1* gene under no IPTG induction; PK-Pa^+^, complementary strain PK-Pa under IPTG induction.

Rhamnolipids are secreted surfactant glycolipids formed by acylation of L-rhamnose using 3-hydroxydecanoyl-ACP (Zhu and Rock, 2008). Therefore, the levels of rhamnolipids produced by *P. aeruginosa* mutant strains were examined. First, TLC was used to analyze the rhamnolipids produced by *P. aeruginosa* strains. The amount of rhamnolipids in *acpP1* mutant strain PA-A1 was decreased, but complementation with plasmid pSRK-Pa, in which the wild-type *acpP1* gene was cloned into pSRK-Km, restored the ability to produce rhamnolipids to the level of that in wild type strain PAO1 (**Figure 4A**). Quantification of rhamnolipids by colorimetric detection of rhamnose showed a twofold decrease in the *acpP1* mutant PA-A1, while the amount of rhamnolipids produced by complemented strain PK-Pa (PA-A1/pSRKPa) was up to 80% of that of the wild type strain PAO1 (Supplementary Figure S6). We also analyzed the 3-hydroxyacids composition of the rhamnolipids produced by *acpP1* mutant strains. The data showed that, although the same fatty acid species were present in PA-A1 as in the wild type PAO1, the 3-hydroxyacids were quantitatively reduced among the rhamnolipids produced by PA-A1 (**Figure 4B**). Next, we investigated the amount of rhamnolipids produced by *P. aeruginosa acpP2* or *acpP3* mutant strains. The level of rhamnolipids produced by PA-A2, PA-A3, or PA-A23 was almost the same as that in strain PAO1. In strain PA-A1, disruption of a single *acpP2* or single *acpP3*, or disruption of both *acpP2* and *acpP3* genes, did not cause the reduction in rhamnolipids seen following replacement of *P. aeruginosa acpP1* with the *E. coli acpP* gene (data not shown).

Pyoverdine is the dominant siderophore of *P. aeruginosa* and is assembled from tetradecanoyl-ACP (Quadri et al., 1999). Secretion of pyoverdine by *P. aeruginosa acpPs* mutant strains was examined by culture on LB–chrome azurol S (CAS) indicator plates. Zones around the cultures that change from blue to yellow–orange indicate where the siderophores have sequestered Fe^3+^ away from the blue CAS-Fe^3+^ complex. Both wild type strain PAO1 and mutant strain PA-A1 generated similar prominent clear zones, which suggested that replacement of *acpP1* with *E. coli acpP* did not affect siderophore secretion (**Figure 4C**). Moreover, the yellow–orange halo around mutant strains PA-A2, PA-A3, and PA-A23 was as large as that around strain PAO1. In addition, on a background of strain PA-A1, mutation of *acpP2* or *acpP3* did not reduce siderophore secretion by *P. aeruginosa* (data not shown).

On the basis of the above results, we conclude that neither *acpP2* nor *acpP3* is involved in fatty acid synthesis in *P. aeruginosa* and that they have no function in the production of various exo-products. Furthermore, although *E. coli* ACP replaces the function of *P. aeruginosa* AcpP1 in fatty acid synthesis, *P. aeruginosa* AcpP1 is required for the production of some exo-products, including three acylated quorum-sensing signal molecules and rhamnolipids.

### Replacement of *acpP1* with *E. coli acpP* Abolished *P. aeruginosa* Swarming Motility

**FIGURE 5:**
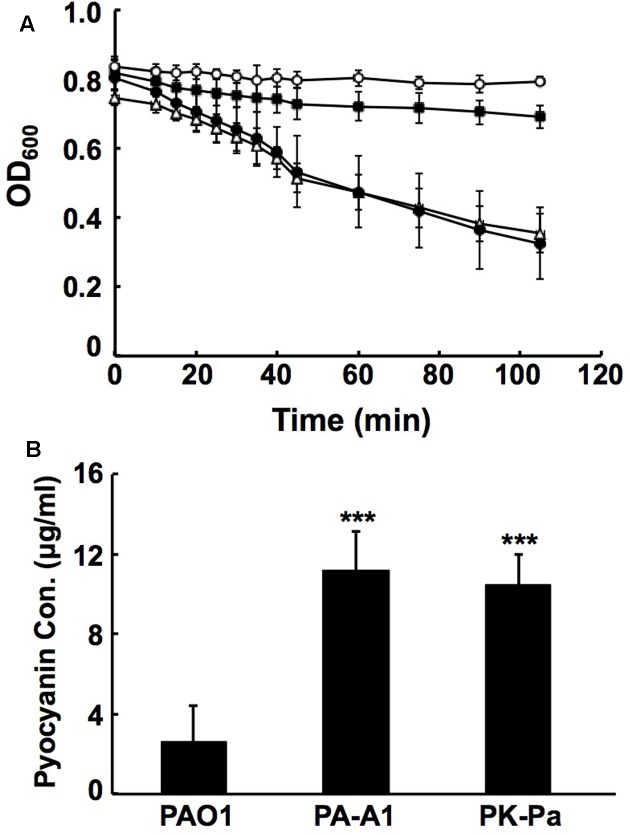
Analysis of LasA activities and pyocyanin production in *P. aeruginosa acpP1* mutant strains. **(A)** LasA protease activities in culture supernatants of *P. aeruginosa* strains. Empty circle indicates boiled *Staphylococcus aureus* cells; filled square indicates boiled *S. aureus* cells containing culture supernatants of *P. aeruginosa* mutant strain PA-A1; filled circle indicates boiled *S. aureus* cells containing culture supernatants of *P. aeruginosa* PAO1; empty triangle indicates boiled *S. aureus* cells containing culture supernatants of complementary strain PK-Pa. **(B)** Pyocyanin in culture supernatants of *P. aeruginosa* strains. Data are the mean ± standard deviation of triplicate measurements. Pair-wise comparisons were made between wild type strain PAO1 and mutant strain PA-A1 or complementary strain PK-Pa by Student’s *t*-test. ^∗∗∗^Highly significant difference, *P* < 0.01.

The QS systems, including *rhl*, *las,* and *PQS*, not only control the production of many virulence factors by *P. aeruginosa* (such as LasA/LasB, rhamnolipid, procyanin, and others), but are also involved in the regulation of *P. aeruginosa* motility (Jimenez et al., 2012). Replacement of *acpP1* by *E. coli acpP* caused *P. aeruginosa* to reduce the production of QS signal molecules. Therefore, the mutant strain PA-A1 was expected to have reduced production of some virulence factors and attenuated motility. To confirm this, we first tested the production of LasA, LasB, and procyanin. LasA protease activity was determined by measuring the ability of *P. aeruginosa* culture supernatants to lyse boiled *S. aureus* cells. The data showed that the activity of LasA in mutant strain PA-A1 was much lower than that in wild type strain PAO1, but complementation strain PK-Pa (PA-A1/pSRK-Pa) increased LasA to the level of that in wild type strain PAO1 (**Figure 5A**). The elastase proteolytic activity (LasB) was measured using the elastin Congo red assay. In comparison with that in wild type strain PAO1, the activity of LasB was decreased twofold in the PA-A1 strain, while the activity of LasB in complemented strain PK-Pa (PA-A1/pSRK-Pa) was up to 80% of that in wild type strain PAO1 (data not shown). However, procyanin production by mutant strain PA-A1 was significantly increased (*P* < 0.005) in comparison with wild type strain PAO1, and plasmid pSRK-Pa did not restore the ability of strain PA-A1 to produce procyanin to the level of that in the wild type strain, indicating that other factors may be involved in the regulation of the production of procyanin in mutant strain PA-A1 (**Figure 5B**).

**FIGURE 6:**
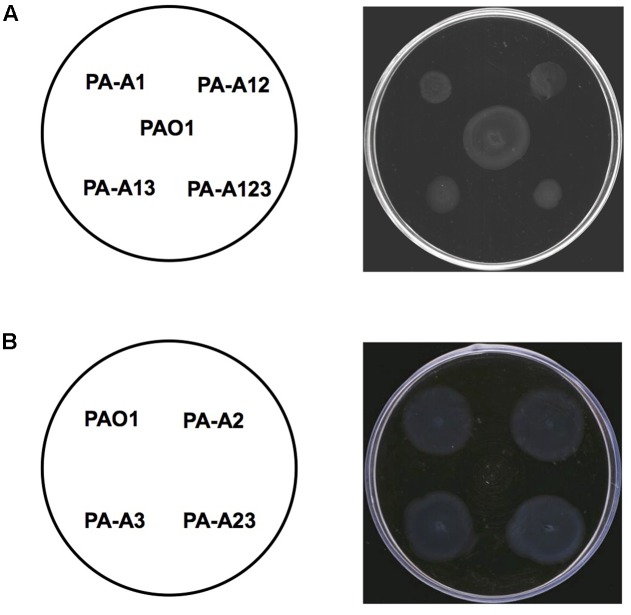
Twitching motility of *P. aeruginosa* strains. **(A)** Twitching motility patterns of *P. aeruginosa* *acpP1* mutant strains after incubation at 37°C for 24 h. PAO1, *P. aeruginosa* wild type strain; PA-A1, *P. aeruginosa acp1*::Ec *acpP* single mutant strain; PA-A12, *P. aeruginosa acp1*::Ec *acpP* *acp2*::Gm^r^ double mutant strain; PA-A13, *P. aeruginosa acp1*::Ec *acpP* *acp3*::Gm^r^ double mutant strain; PA-A123, *P. aeruginosa acp1::*Ec *acpP acp2::*Tc^r^ *acp3::*Gm^r^ triple mutant strain. **(B)** Twitching motility patterns of *P. aeruginosa* *acpPs* mutant strains after incubation at 37°C for 24 h. PAO1, *P. aeruginosa* wild type strain; PA-A2, *P. aeruginosa acpP2*::Gm^r^ single mutant strain; PA-A3, *P. aeruginosa* *acpP3*::Gm^r^ single mutant strain; PA-A23, *P. aeruginosa* *acp2*::Tc^r^ *acp3*::Gm^r^ double mutant strain.

We tested three types of motility: swimming, swarming, and twitching, in mutant strain PA-A1. The swimming pattern of mutant stain PA-A1 was almost the same as that of wild type strain PAO1, which suggested that replacement of *acpP1* with *E. coli* *acpP* did not affect the swimming motility of *P. aeruginosa* (data not shown). However, replacement of *acpP1* by *E. coli acpP* reduced the twitching ability of *P. aeruginosa*. The twitching pattern of mutant PA-A1 was much smaller than that of the PAO1 strain (**Figure 6A**). We also tested the effect of mutation of *acpP2* or *acpP3* on twitching motility. The data showed that neither single mutation of *acpP2* or *acpP3* nor double mutation of *acpP2* and *acpP3* affected *P. aeruginosa* twitching motility (**Figures 6A,B**).

**FIGURE 7:**
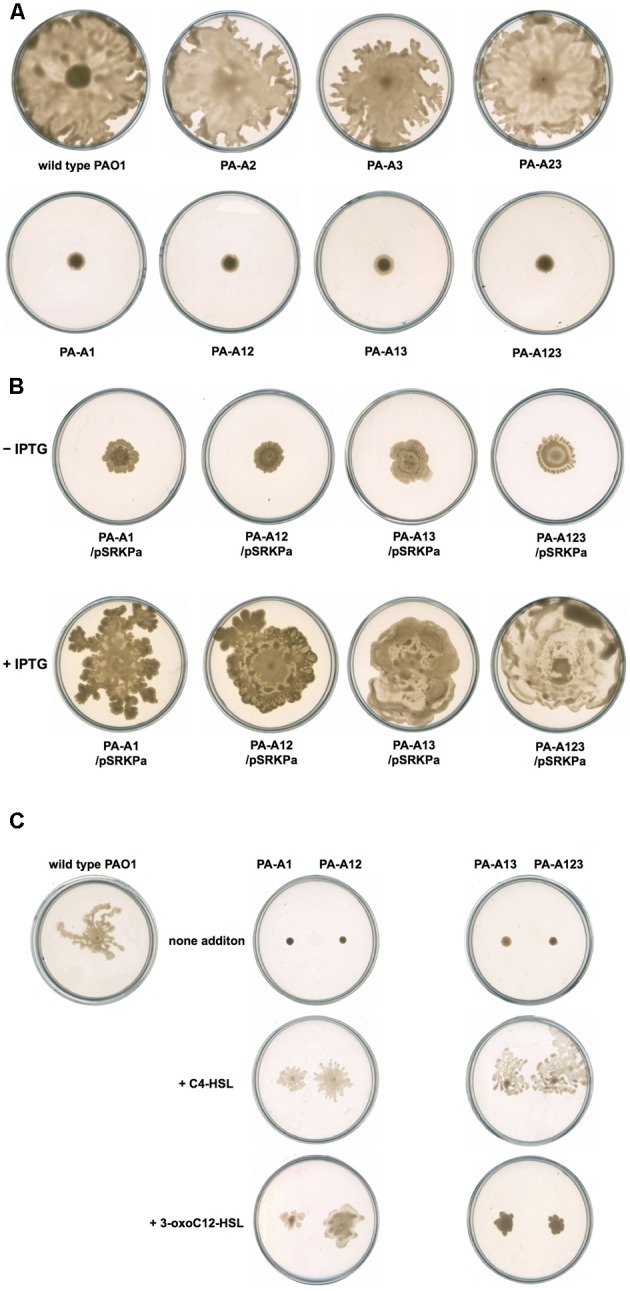
Swarming motility of *P. aeruginosa* strains. **(A)** Swarming motility patterns of *P. aeruginosa acpPs* mutant strains. **(B)** Swarming motility patterns of *P. aeruginosa acpP1* mutant strains carried plasmid pSRK-Pa. **(C)** Effect of exogenous AHLs on swarming motility of *P. aeruginosa acpP1* mutant strains. PAO1, *P. aeruginosa* wild type strain; PA-A1, *P. aeruginosa acp1*::Ec *acpP* single mutant strain; PA-A2, *P. aeruginosa acpP2*::Gm^r^ single mutant strain; PA-A3, *P. aeruginosa* *acpP3*::Gm^r^ single mutant strain; PA-A12, *P. aeruginosa acp1*::Ec *acpP* *acp2*::Gm^r^ double mutant strain; PA-A13, *P. aeruginosa acp1*::Ec *acpP* *acp3*::Gm^r^ double mutant strain; PA-A23, *P. aeruginosa* *acp2*::Tc^r^ *acp3*::Gm^r^ double mutant strain; PA-A123, *P. aeruginosa acp1::*Ec *acpP acp2::*Tc^r^ *acp3::*Gm^r^ triple mutant strain.

Next, we investigated the swarming motility of mutant strain PA-A1. Replacement of *acpP1* by *E. coli acpP* caused *P. aeruginosa* swarming to fail. On semisolid plates (containing 0.5% agar), wild type strain PAO1 formed a typical swarming pattern, while mutant strain PA-A1 only formed a small circular lawn (**Figure 7A**). However, when complemented with pSRK-Pa, the ability of PA-A1 to form a typical swarming pattern was restored (**Figure 7B**). We also noticed that mutant strains PA-A2, PA-A3, and PA-A23 could form a typical swarming pattern, indicating that mutation of *acpP2* or *acpP3* did not affect *P. aeruginosa* swarming motility (**Figures 7A,B**). Because replacement of *acpP1* by *E. coli acpP* causes *P. aeruginosa* to reduce the production of QS signal molecules, we wondered if exogenous addition of AHLs signals could restore the ability of mutant strain PA-A1 to swarm on semisolid plates. To test this hypothesis, we added 2 μM C_4_-HSL or 3-oxo-C_12_-HSL to semisolid plates. After 24 h of incubation, on both types of semisolid plate, the swarming motility of mutant strain PA-A1 was restored (**Figure 7C**), even though the swarming patterns were still smaller than that of the wild type strain PAO1. This indicated that reduction of the AHLs signal was the main factor that caused *P. aeruginosa* to fail to swarm.

## Discussion

The *P. aeruginosa* genome contains three open reading frames, PA2966, PA1869, and PA3334, that encode putative ACP: AcpP1, AcpP2, and AcpP3, respectively. Alignments of *P. aeruginosa* AcpPs with the *E. coli* ACP showed that AcpP1, AcpP2, and AcpP3 share 87, 45, and 30% identical residues with *E. coli* ACP, respectively, and the serine residue that is presumed to be phosphopantetheinylated is conserved among these AcpPs (Raychaudhuri et al., 2005). Our *in vitro* assays showed that these apo-ACPs were successfully phosphopantetheinylated by *P. aeruginosa* PcpS and that all holo-forms of these proteins could be acylated by *V*. *harveyi* AasS, which suggested that all these proteins may be ACP. However, only AcpP1 could be used as a substrate for the synthesis of fatty acids *in* *vitro*, catalyzed by *P. aeruginosa* cell-free extracts, and only *acpP1* could restore growth in the *E. coli acpP* mutant strain CY1877. Moreover, *acpP1* is essential for the growth of *P. aeruginosa* and cannot be deleted, but disruption of *acpP2* or *acpP3* in *P. aeruginosa* mutant strains allowed them to grow as well as the wild type strain, and mutation of the *acpP2* or *acpP3* gene did not cause changes in the fatty acid composition in *P. aeruginosa* cells. These findings confirm that only *P. aeruginosa* AcpP1 functions in fatty acid biosynthesis, and that *acpP2* and *acpP3* do not play roles in the fatty acid synthetic pathway.

Replacement of *P. aeruginosa* *acpP1* with *E. coli acpP* did not affect *P. aeruginosa* growth, and the fatty acid composition of this mutant strain was almost the same as that of the wild type strain, which suggests that *E. coli* ACP may be a good substrate for *P. aeruginosa* fatty acid synthetic enzymes. However, the production of some exo-products, including three acylated quorum-sensing signal molecules (C_4_-HSL, 3-oxo-C_12_-HSL, and PQS) and rhamnolipids, was reduced. It is well known that the precursors of these products come from intermediates in the *P. aeruginosa* fatty acid synthetic pathway (Hoang et al., 2002). However, the *E. coli* strain possesses no enzymes involved in the production of these exo-products and therefore does not produce the above exo-products. Thus, even though *E. coli* ACP can be used as a good substrate for *P. aeruginosa* fatty acid synthetic enzymes to produce acyl-ACP intermediates, *P. aeruginosa* enzymes that are responsible for the production of the above exo-products fail to recognize intermediates carried by *E. coli* ACP as a good substrate, which causes reduced production of these exo-products in *P. aeruginosa*.

Replacement of *acpP1* with *E. coli acpP* also abolished *P. aeruginosa* swarming motility. However, addition of exogenous C_4_-HSL or 3-oxo-C_12_-HSL could restore the swarming motility of the *P. aeruginosa* mutant PA-A1 strain, which was consistent with the reduction in the amount of C_4_-HSL or 3-oxo-C_12_-HSL produced by mutant PA-A1. A similar phenomenon was also observed when we studied the *P. aeruginosa* *fabV* mutant: the *fabV* mutant showed reduced production of C_4_-HSL and 3-oxo-C_12_-HSL signals, and failed to swarm, but addition of exogenous C_4_-HSL or 3-oxo-C_12_-HSL restored the swarming motility of the *fabV* mutant strain (Huang et al., 2016). These findings suggest that an insufficient number of QS signals was the main factor that caused *P. aeruginosa* to fail to swarm, and that the QS system plays an important role in *P. aeruginosa* swarming motility.

*P. aeruginosa* produces two siderophores, pyoverdine, and pyochelin, which are involved in iron acquisition and are required for virulence (Moore and Flaws, 2011). Replacement of *acpP1* with *E. coli acpP* did not affect the ability of *P. aeruginosa* to secrete siderophores. This indicated that *P. aeruginosa* ACPs are not involved in the synthesis of these two siderophores. Indeed, pyoverdine and pyochelin are produced by non-ribosomal peptide synthases (Quadri et al., 1999; Barekzi et al., 2004). For pyoverdine, the associated carrier proteins are PvdD, PvdI, and PvdJ, while pyochelin synthesis requires the PchE and PchF carrier proteins (Barekzi et al., 2004).

Quorum-sensing signaling is necessary for production of the phenazine secondary metabolite pyocyanin (Jimenez et al., 2012). However, although replacement of *acpP1* with *E. coli acpP* reduced the ability of *P. aeruginosa* to produce quorum-sensing signals, the mutant strain PA-A1 showed significantly increased production of pyocyanin in comparison with the wild type strain. Recent studies have shown that an RpoS-dependent sRNA RgsA represses the expression of *P. aeruginosa* AcpP1 at the post-transcriptional level by base-pairing interactions between RgsA and *acpP1* mRNA (Lu et al., 2016). That report also showed that overexpression of full-length RgsA in *P. aeruginosa* could increase the production of pyocyanin. Therefore, on the basis of these findings, we speculate that replacement of *acpP1* with *E. coli acpP* caused *P. aeruginosa* to fail to produce *acpP1* mRNA, and increased free RgsA in *P. aeruginosa* cells. Under the action of RgsA, *P. aeruginosa* increased the production of pyocyanin. However, the details of the mechanism need further study.

In *P. aeruginosa*, C_4_-HSL, a quorum-sensing signal of the Rhl signaling system, is produced from *S*-adenosyl-methionine and butyryl-ACP, a process catalyzed by RhlI (Jimenez et al., 2012). Previous *in vitro* studies (Raychaudhuri et al., 2005) showed that AcpP2 molecules are good substrates for RhlI. However, following disruption of the *acpP2* gene in the *P. aeruginosa* genome, mutant strain PA-A2 did not show a reduced level of C_4_-HSL. Moreover, although replacement of *acpP1* with *E. coli acpP* caused the amount of C_4_-HSL produced by *P. aeruginosa* to decrease, the double mutant strain PA-A12, in which *acpP1* was replaced with *E. coli acpP* and *acpP2* was disrupted, produced the same level of C_4_-HSL as that produced by the single mutant strain PA-A1. All these findings indicate that AcpP2 is not a good substrate for RhlI *in vivo*. Our studies also confirmed that AcpP2 was not involved in the production of 3-oxo-C_12_-HSL and rhamnolipids. Therefore, the functions of AcpP2 in *P. aeruginosa* need further investigation.

Disruption of *acpP3* did not affect growth, the fatty acid composition of phospholipids or production of some exo-products in *P. aeruginosa*. This should be expected, because *acpP3* is located in a gene cluster that encodes an unknown secondary metabolite synthetic pathway (Quadri et al., 1999).

## Author Contributions

J-CM cloned acpPs gene, and constructed expression vectors. DC constructed acpP mutants, purified ACP proteins. Y-QW tested the production of QS signals and motility in mutant strains. W-BZ analyzed fatty acids composition of acpP strains and analyzed the function of ACPs *in vitro*. J-CM also participated in the design of the study and helped to draft the manuscript. H-HW conceived of the study, and participated in its design and coordination and helped to draft the manuscript. All authors read and approved the final manuscript.

## Conflict of Interest Statement

The authors declare that the research was conducted in the absence of any commercial or financial relationships that could be construed as a potential conflict of interest.
