# Supplementary material for: Only Acyl Carrier Protein 1 (AcpP1) Functions in Pseudomonas aeruginosa Fatty Acid Synthesis
Source: Front Microbiol. 2017 Nov 10;8:2186. doi: 10.3389/fmicb.2017.02186 (PMC5686131; doi:10.3389/fmicb.2017.02186)
Supplement: Supplementary file 1 [file Data_Sheet_1.docx]

**Only acyl carrier protein 1 (AcpP1) of *Pseudomonas aeruginosa* functions in fatty acid synthesis**

Jin-Cheng Ma^1^, Yun-Qi Wu^2^, Dan Cao^3^, Wen-Bin Zhang^1^, Hai-Hong Wang^1^*

^1^Guangdong Provincial Key Laboratory of Protein Function and Regulation in Agricultural Organisms, College of Life Sciences, South China Agricultural University, Guangzhou, Guangdong 510642, China

^2^Department of Chemistry and Biomolecular Sciences, Macquarie University, North Ryde, NSW, Australia 2109

^3^Forensic Science Center of Qingyuan, Qingyaun Public Security Department, Qingyaun, Guangdong 511500, China

**Supplementary Tables**

**Table S1. Bacterial strains and plasmids used in this study**

| Bacterial strains | Relevant characteristics*^a^* | Source |
| --- | --- | --- |
| *E. coli* |  |  |
| DH-5α | F^-^ *deoR* *endA1 gyrA96 hsdR17*(r_K_^-^m_K_^+^) *recA1* *relA1* *supE*44 *thi-1* Δ(*lacZYA-argF*)*U*169(φ80*lacZ*ΔM15) | Laboratory collection |
| BL21 (DE3) | F^-^ *dcm ompT* *hsdS* (*r_B_*^-^ *m_B_*^-^) *gal* (λDE3) | Laboratory collection |
| S17-1 | F^-^ *thi pro* *hsdR* [RP4-2 Tc::Mu Km::Tn7 (Tp Sm)] | Laboratory collection |
| CY1877 | *E.coli* MG1655 *acpP*::Cm^r^ carrying pBAD24-acpP | Laboratory collection |
| *P. aeruginosa* | |  |
| PAO1 | Wild-type strain | Laboratory collection |
| PA-A1 | PAO1, *acpP1*::*EcacpP* | This study |
| PA-A2 | PAO1, *acpP2*::Gm^r^ | This study |
| PA-A3 | PAO1, *acpP3*::Gm^r^ | This study |
| PA-A12 | PAO1, *acpP1*::*EcacpP acpP2*::Gm^r^ | This study |
| PA-A13 | PAO1, *acpP1*::*EcacpP acpP3::*Gm^r^ | This study |
| PA-A23 | PAO1, *acpP2*::Tc^r^ *acpP3::*Gm^r^ | This study |
| PA-A123 | PAO1, *acpP1*::*EcacpP acpP2*::Tc^r^ *acpP3*::Gm^r^ | This study |
| PK-Pa | PAO1, *acpP1*::*EcacpP* carried plasmid pSRKPa | This study |
| Plasmids |  |  |
| pMD19 | Amp^r^, TA cloning vector | Takara |
| pBAD24m | Amp^r^; NcoI site of expression vector pBAD24 changed to an NdeI site | (7) |
| pET-28(b) | Km^r^; expression vector | Novagen |
| pET-30(a) | Km^r^; expression vector | Novagen |
| pBluescript SK(+) | Amp^r^; clone and expression vector | Laboratory collection |
| pTac85 | Amp^r^; expression vector | (4) |
| pK18mobscaB | Km^r^; *sacB*-based gene replacement vector | (5) |
| p34s-Gm | Amp^r^; Gm resistance cassette-carrying vector | (1) |
| p34s-Tc | Amp^r^; Tc resistance cassette-carrying vector | (1) |
| pSRK-Km | Km^r^, broad-host-range expression vector containing *lac* promoter and *lacI^q^*, *lacZα^+^* | (3) |
| pHSG399 | Cm^r^; clone and expression vector | (6) |
| pYFJ84 | *V. hareyi aasS* cloned into the NdeI and BamHI sites of pET16(b) | (2) |
| pBAD24m- *acpP1* | Amp^r^; The PCR fragment of *acpP1* from *P.aeruginosa* genome digested by NdeI and HindIII was cloned into the same sites of pBAD24m | This study |
| pBAD24m- *acpP2* | Amp^r^; The PCR fragment of *acpP2* from *P.aeruginosa* genome digested by NdeI and HindIII was cloned into the same sites of pBAD24m | This study |
| pBAD24m- *acpP3* | Amp^r^; The PCR fragment of *acpP3* from *P.aeruginosa* genome digested by NdeI and HindIII was cloned into the same sites of pBAD24m | This study |
| pCD4 | Km^r^; The PCR fragment of *acpP1* from pBAD24m-*acpP1* digested by XbaI and HindIII was cloned between XbaI and HindIII sites of pET-28 (b) | This study |
| pCD5 | Km^r^; The PCR fragment of *acpP2* from pBAD24m-*acpP2* digested by XbaI and HindIII was cloned between XbaI and HindIII sites of pET-28 (b) | This study |
| pCD6 | Km^r^; The *acpP3* fragment of pBAD24m-*acpP3* digested by NdeI and HindIII was cloned between NdeI and HindIII sites of pET-30 (a) | This study |
| pCD8 | Amp^r^; The XbaI-HindIII-digested fragment from pCD04 was cloned between the same sites of pBluescript SK(+) | This study |
| pCD9 | Amp^r^; The XbaI-HindIII-digested fragment from pCD05 was cloned between the same sites of pBluescript SK(+) | This study |
| pCD10 | Amp^r^; The XbaI-HindIII-digested fragment from pCD06 was cloned between the same sites of pBluescript SK(+) | This study |
| pCD1 | Amp^r^; The PCR fragment of *acpP1* from pCD8 digested by BamHI and KpnI was cloned into the same sites of pTac85 | This study |
| pCD2 | Amp^r^; The PCR fragment of *acpP2* from pCD9 digested by BamHI and KpnI was cloned into the same sites of pTac85 | This study |
| pCD3 | Amp^r^; The PCR fragment of *acpP2* from pCD10 digested by BamHI and SalI was cloned into the same sites of pTac85 | This study |
| pCD15 | Km^r^; The PCR fragment containing *P.aeruginosa pcpS* digested by NdeI and HindIII was between the same sites of pET28(b) | This study |
| pSRKPa | Km^r^; The *P.aeruginosa acpP1* was cloned between NdeI and HindIII sites of pSRK-Km | This study |
| pCD16 | Amp^r^; The 1.3-kb PCR fragment of *P.aeruginosa* genome containing *acpP1* digested by BamHI and HindIII was cloned between the same sites of pBluescript SK(+) | This study |
| pCD17 | Amp^r^ Gm^r^; Gm resistant cassette of p34s-Gm digested by EcoRI was cloned into the same site of pCD16 | This study |
| pCD18 | Km^r^ Gm^r^; The BamHI-HindIII fragment from pCD17 was cloned between the same sites of pK18mobscaB | This study |
| pCD19 | Cm^r^; The 1.0-kb PCR fragment of *P.aeruginosa* genome containing *acpP2* digested by EcoRI and HindIII was cloned between the same sites of pHSG399 | This study |
| pCD20 | Cm^r^ Gm^r^; Gm resistant cassette of p34s-Gm digested by XbaI was cloned into the same site of pCD19 | This study |
| pCD21 | Km^r^ Gm^r^; The EcoRI-HindIII fragment from pCD20 between the same sites of pK18mobscaB | This study |
| pCD22 | Km^r^ Tc^r^; Tc resistant cassette of p34s-Tc digested by BamHI was cloned into the same site of pCD21 | This study |
| pCD23 | Cm^r^; The 886-bp PCR fragment of *P.aeruginosa* genome containing *acpP3* digested by EcoRI and HindIII was cloned between the same sites of pHSG399 | This study |
| pCD24 | Cm^r^ Gm^r^; Gm resistant cassette of p34s-Gm digested by SalI was cloned into the same site of pCD23 | This study |
| pCD25 | Km^r^ Gm^r^; The EcoRI-HindIII fragment from pCD24 was cloned between the same sites of pK18mobscaB | This study |
| pCD26 | Amp^r^; pCD17 was digested with EcoRI and was ligated itself | This study |
| pCD27 | Km^r^; The BamHI-HindIII fragment from pCD26 was cloned between the same sites of pK18mobscaB | This study |
| pCD28 | Km^r^ Gm^r^; Gm resistant cassette of p34s-Gm digested by BamHI was cloned into the same site of pCD27 | This study |
| pCD29 | Amp^r^; The PCR fragment of acpP1up-EcacpP-acpP1down was ligated to pMD19 | This study |
| pCD30 | Km^r^; The BamHI-HindIII fragment from pCD29 was cloned between the same sites of pK18mobscaB | This study |
| pCD31 | Km^r^ Gm^r^; Gm resistant cassette of p34s-Gm digested by BamHI was cloned into the same site of pCD30 | This study |

**References**

1. Dennis, J. J., and G. J. Zylstra. 1998. Plasposons: modular self-cloning minitransposon derivatives for rapid genetic analysis of gram-negative bacterial genomes. Appl Environ Microbiol 64:2710-2715.
2. Jiang, Y., C. H. Chan, and J. E. Cronan. 2006. The soluble acyl-acyl carrier protein synthetase of Vibrio harveyi B392 is a member of the medium chain acyl-CoA synthetase family. Biochemistry 45:10008-10019.
3. Khan, S. R., J. Gaines, R. M. Roop, 2nd, and S. K. Farrand. 2008. Broad-host-range expression vectors with tightly regulated promoters and their use to examine the influence of TraR and TraM expression on Ti plasmid quorum sensing. Appl Environ Microbiol 74:5053-5062.
4. Marsh, P. 1986. Ptac-85, an E. coli vector for expression of non-fusion proteins. Nucleic Acids Res 14:3603.
5. Schafer, A., A. Tauch, W. Jager, J. Kalinowski, G. Thierbach, and A. Puhler. 1994. Small mobilizable multi-purpose cloning vectors derived from the Escherichia coli plasmids pK18 and pK19: selection of defined deletions in the chromosome of Corynebacterium glutamicum. Gene 145:69-73.
6. Takeshita, S., M. Sato, M. Toba, W. Masahashi, and T. Hashimoto-Gotoh. 1987. High-copy-number and low-copy-number plasmid vectors for lacZ alpha-complementation and chloramphenicol- or kanamycin-resistance selection. Gene 61:63-74.
7. Zhu, L., J. Lin, J. Ma, J. E. Cronan, and H. Wang. 2010. Triclosan resistance of Pseudomonas aeruginosa PAO1 is due to FabV, a triclosan-resistant enoyl-acyl carrier protein reductase. Antimicrob Agents Chemother 54:689-698.

**Table S2. Sequences of the PCR primers used**

| Name | Sequences (5'→3') |
| --- | --- |
| AcpP1 NdeI | AAACAAcatatgAGCACCATCGAAGAACGC |
| AcpP1 HindIII | CGACGAaagcttATTGCTGGTGAGCAACG |
| AcpP2 NdeI | CCGCAAcatatgGACGACATCGAGACCAG |
| AcpP2 HindIII | CCAGGGaagcttAGGTCGGCACGGCTTCC |
| AcpP3 NdeI | GGAAGAcatatgCCCAACGACATGGAAG |
| AcpP3 HindIII | TTCGCTaagcttAGGCGGCGCGGTGCTTC |
| AcpPs Xba I | TTTTGGtctagaAGGAGGAATTCCATATG |
| AcpP1 BamHI | ACATATggatccATGAGCACCATCGAAGAACG |
| AcpP2 BamHI | ACATATggatccATGGACGACATCGAGACCAG |
| AcpP3 BamHI | ACATATggatccATGCCTAACGACATGGAAGAC |
| PcpS NdeI | ACCGGCcatatgCGCGCCATGAACGACCGTCTC |
| PcpS HindIII | ATATCTaagcttGATCAGGCGCCGACCGCCAC |
| AcpP1up HindIII | ATAATCaagcttTGCGCATGAAAGACGACGAG |
| AcpP1down BamHI | CGCCGAggatccAACCCATGGATACGCCGATAC |
| AcpP1up HindIII | CGAGCGaagcttGGGCCTGTCGTTGCGTTTGC |
| AcpP1down EcoRI | GCCTCGgaattcCGCCGCGAGGAAAATTCGGCCTC |
| AcpP1up XbaI | CTTCCAtctagaTGACCAGTTCGACTACCTCG |
| AcpP1down XbaI | TCGGCGtctagaATCGCCGATGACGATGCGGAAC |
| Acp3up HindIII | CCAGGCaagcttGCAGATGAGCGACTCGGTGC |
| Acp3down EcoRI | AGGTCGgaattcAGATAGCGGGCATTGAGCAG |
| Gmup EcoRI | GAGCTCgaattcACATAAGCCTGTTCGGTTC |
| Gmdown EcoRI | GAGCTCgaattcGCCGCGGCGTTGTGAC |
| Pa acpP up | ACCAGGCGTAAGAATTCGAGACCGAAATCCC |
| Pa acpP down | TAGTGCTCATACCTTGTTTTCACTCCTATGG |
| Ec acpP up | AAAACAAGGTATGAGCACTATCGAAGAACG |
| Ec acpP down | TCTCGAATTCTTACGCCTGGTGGCCGTCGATG |
|  |  |
| AcpPup2 | TGGTCATCGGCACCGCGACCAGCGCGTC |
| AcpPdown2 | CGGCCTCGCCATAGGCGATGTTGCGAGC |
| Acp1up2 | TATCCGGCTCCAGCCCCGGTGCTGATGG |
| Acp1down2 | ATCACGACGCGCCAGCTCGGCCTCGTCG |
| Acp3up2 | GGTGGACACTTTCAGCCATACCCTCGAC |
| Acp3down2 | TCGCGGAAGTTGCCCAGCAGGTTGAACG |

Note: lower case letters show the restriction sites.

**Table S3. Fatty acid composition of total lipid extracts from *P. aeruginosa* *acpPs* mutant strains** *^a^*

| Fatty acid(%) | *P. aeruginosa* strains | | | | |
| --- | --- | --- | --- | --- | --- |
|  | PAO1 | PA-A12 | PA-A13 | PA-A23 | PA-A123 |
| *n*-C_10:0_-3-OH*^b^* | 3.78 ± 0.69 | 4.80 ± 0.70 | 4.03 ± 0.43 | 4.73 ± 0.42 | 5.47 ± 0.24 |
| *n*-C_12:0_-3-OH | 6.60 ± 0.76 | 6.71 ± 0.05 | 5.58 ± 0.04 | 6.23 ± 0.87 | 7.40 ± 0.56 |
| n-C_16:1_ | 11.26 ± 0.50 | 10.42 ± 0.88 | 16.35 ± 0.56 | 12.93 ± 0.37 | 14.49 ± 0.98 |
| n-C_16:0_ | 34.49 ± 0.36 | 34.80 ± 0.37 | 31.91 ± 0.99 | 33.35 ± 1.16 | 32.40 ± 0.88 |
| n-C_18:1_ | 35.85 ± 1.91 | 34.02 ± 1.05 | 36.57 ± 0.87 | 38.59 ± 0.74 | 34.09 ± 0.96 |
| n-C_18:0_ | 8.01 ± 0.35 | 9.24 ± 1.92 | 5.57 ± 0.16 | 4.17 ± 0.41 | 6.15 ± 0.27 |

*^a^* Cells were grown in LB medium for 12 h at 37°C. Total lipids were extracted and transesterified to fatty acid methyl esters, and products identified by GC-MS. Values are percentages of total fatty acids and are means ± standard deviations of three independent experiments.

***^b^*** n-C_10:0_ 3-OH, 3-hydroxyldecanoic acid; n-C_10:0_ 3-OH, 3-hydroxyldodecanoic acid; n-C_16:1_, *cis*-9-hexadecenoic acid; n-C_16:0_, hexadecanoic acid; n-C_18:1_, *cis*-11-octadecenoic acid; n-C_18:0_, octadecanoic acid.

**Supplementary Figures**

**Figure S1**

**Figure S1** A. Strategy for isolation of *P. aeruginosa* *acpPs* mutant strains. B. Genetic organization of the *acpPs* region in PAO1 (a) or *acpPs* strains (b). C. Colony PCR analysis of mutant strains. Lane 1 and 3, PCR products of strain PAO1; lane 2, PCR products of PA-A2; lane 4, PCR products of PA-A3. Abbreviations: CH, chromosome; Up, upstream fragment of *acpPs*; Dn, downstream fragment of *acpPs*, Gm, gentamicin resistant cassette; pCDs, suicide plasmids used for disruption of *P. aeruginosa acpPs*.

**Figure S2**

**Figure S2** Strategy for deletion of *P. aeruginosa acpP1*. Abbreviations: CH, chromosome; Up, upstream fragment of *acpP1*; Dn, downstream fragment of *acpP1*, Gm, gentamicin resistant cassette; pCD28, suicide plasmids used for disruption of *P. aeruginosa acpP1*.

**Figure S3**

**Figure S3** A. Strategy for replacement of *P. aeruginosa acpP1* with *E.coli acpP*. B. Genetic organization of the *acpP1* region in PAO1 (a) or *acpP1*::*EcacP* (b). C. Colony PCR analysis of *P. aeruginosa* strains in (B). Abbreviations: CH, chromosome; Up, upstream fragment of *acpP1*; Dn, downstream fragment of *acpP1*, Gm, gentamicin resistant cassette; pCD31, suicide plasmids used for replacement of *P. aeruginosa acpP1* with *E.coli acpP*; *EcacpP*, *E.coli acpP*.

**Figure S4**

**Figure S4** Colony PCR analysis of *P. aeruginosa acpPs* mutant strains. A. Colony PCR analysis of *P. aeruginosa acpP1::EcacpP acpP2::Gm* mutant strain. B. A. Colony PCR analysis of *P. aeruginosa acpP1*::*EcacpP acpP3::Gm* mutant strain. C. A. Colony PCR analysis of *P. aeruginosa acpP2*::Tc *acpP3*::Gm mutant strain. D. A. Colony PCR analysis of *P. aeruginosa acpP1*::*EcacpP acpP2*::Tc *acpP3*::Gm mutant strain. Abbreviations: Gm, gentamicin resistant cassette; Tc, tetracycline resistant cassette; *EcacpP*, *E.coli acpP*.

**Figure S5**

**Figure S5 MALDI-TOF-MS of *P. aeruginosa* three ACPs.** A. Mass spectrum of *P. aeruginosa* AcpP1 purified from *E.coli* cell. B. Mass spectrum of *P. aeruginosa* AcpP1 purified after Phosphopantetheinylatation with *P. aeruginosa* PcpS. C. Mass spectrum of *P. aeruginosa* AcpP2 purified from *E.coli* cell. D. Mass spectrum of *P. aeruginosa* AcpP2 purified after Phosphopantetheinylatation with *P. aeruginosa* PcpS. E. Mass spectrum of *P. aeruginosa* AcpP3 purified from *E.coli* cell. F. Mass spectrum of *P. aeruginosa* AcpP3 purified after Phosphopantetheinylatation with *P. aeruginosa* PcpS.

**Figure S6**

**Figure S6 Analysis production of rhamnolipids in *P. aeruginosa acpP1* mutant strains by colorimetric detection of rhamnose.** PK-Pa -, *acpP1* mutant PA-A1 carried plasmid pSRKPa grown under no IPTG induction. PK-Pa +, *acpP1* mutant PA-A1 carried plasmid pSRKPa grown under IPTG induction.
